# Simultaneous Optimization of Ultrasound-Assisted Extraction for Flavonoids and Antioxidant Activity of *Angelica keiskei* Using Response Surface Methodology (RSM)

**DOI:** 10.3390/molecules24193461

**Published:** 2019-09-24

**Authors:** Lei Zhang, Yuhuan Jiang, Xuening Pang, Puyue Hua, Xiang Gao, Qun Li, Zichao Li

**Affiliations:** 1 College of Life Sciences, Institute of Advanced Cross-Field Science, Institute of *Angelica keiskei* Health Industry Technology, Qingdao University, Qingdao 266071, China; 2017020832@qdu.edu.cn (L.Z.); 2018025210@qdu.edu.cn (Y.J.); 2018025216@qdu.edu.cn (X.P.); h13399545149@163.com (P.H.); gaoxiang@qdu.edu.cn (X.G.); 2 College of Chemistry and Chemical Engineering, Qingdao University, Qingdao 266071, China; 3 Qingdao Balanson Biotech Co., Ltd., Qingdao 266071, China

**Keywords:** ultrasound-assisted extraction (UAE), *Angelica keiskei*, flavonoids, antioxidant activity, response surface methodology (RSM)

## Abstract

*Angelica keiskei* Koidzumi (*A. keiskei*), as a Japanese edible herbal plant, enjoys a variety of biological activities due to the presence of numerous active compounds, especially flavonoids. This study aims for the optimization of ultrasound-assisted extraction (UAE) for flavonoids in *A. keiskei* and their antioxidant activity by using the response surface methodology (RSM). Single-factor experiments and a four-factor three-level Box–Behnken design (BBD) were performed to explore the effects of the following parameters on flavonoid extraction and antioxidant activity evaluation: ultrasonic temperature (X_1_), ultrasonic time (X_2_), ethanol concentration (X_3_) and liquid–solid ratio (X_4_). The optimum conditions of the combination of total flavonoid content (TFC), 2,2-diphenyl-1-picrylhydrazyl (DPPH) radical scavenging capacity (DPPH-RSC) and ferric-reducing antioxidant power (FRAP) were as follows: X_1_ = 80 °C, X_2_ = 4 min, X_3_ = 78%, X_4_ = 35 mL/g, respectively. The experimental results provide a theoretical basis for the extensive utilization of *A. keiskei* and flavonoids extraction from *A. keiskei* as a potential source of antioxidants.

## 1. Introduction

*Angelica keiskei* Koidzumi (*A. keiskei*), a clumping perennial herb member of the Umbelliferae family, has been described as “Ashitaba” or “Japanese angelica” in Japan [1,2], “Shin-sun cho” or “Myeong-ilyeob” in Korea [3] and “Ming-ri-ye” in China [4]. As a native plant to the island of Hachijojima, the herb is mainly located in the Izu Islands of Japan [5,6], but it has also been cultivated in other Asian countries, including China and South Korea [7]. Particularly, since a successful introduction of *A. keiskei* into China in the 1990s, it has been widely planted in Shandong, Jiangsu, Yunnan, Guizhou and Guangxi provinces to date [8]. The aerial parts of *A. keiskei* has been initially used as ordinary food, especially as vegetables and pickles [9]. Recently, it has gained popularity as a functional or nutritional component used in beverages, daily chemicals and healthcare products, such as tea bags [10], cosmetics [11], capsules of dietary supplements [12], etc. Moreover, it has been reported to possess properties of anti-oxidative [13], anti-bacterial [14], anti-virus [15], anti-osteoporosis [16], anti-tumor [17], anti-hyperlipidemic [18], ameliorate inflammation [19] and prevention of metabolic syndrome [20]. Noticeably, these biological activities are documented and attributed to the presence of varieties of bioactive constituents in *A. keiskei*, such as flavonoids, coumarins, terpenoids, acetylenes, phenolics and other compounds [21].

Flavonoids are universally acknowledged to be a large category of secondary metabolites and widespread in plants, which are divided into eight different categories and contain more than ten thousand kinds of structures [22,23]. So far, scientists have extracted, isolated and identified 56 flavonoids, including 42 chalcones, 8 flavones and 6 flavanones from *A. keiskei* [21]. In recent years, owing to their promising pharmacological properties including coronary heart disease prevention, free radical elimination, cancer prevention and so on, these active compounds have captured great attention among researchers [24]. Oxidants and free radicals are usually generated by two pathways: one is the metabolism of normal cells and the other is the external environment, particularly electromagnetic radiation, air pollution, cigarette smoke and drugs. When the amount of free radicals produced is unbalanced with the amount of free radicals consumed, the accumulation of free radicals in the body can lead to oxidative stress [25]. Moreover, severe oxidative stress can give rise to cardiovascular, neurological and pulmonary diseases, or even cancer. Flavonoids, as a kind of antioxidant, can scavenge excess to free radicals in vivo to avoid oxidative stress and reduce the risk of related diseases [26]. Notably, few reported studies have been focused on the optimization for the flavonoids extraction and their antioxidant activity of *A. keiskei* by using the response surface methodology (RSM).

In general, the solvent extraction process is of great importance to conduct qualitative and quantitative analysis of active ingredients from plants or herbs. However, the composition of the extraction products and the extraction rate of the target products are affected by many factors, particularly extraction factors including method, solvent, time, temperature and liquid–solid ratio [27]. Usually, the traditional liquid–solid extraction methods such as percolation, impregnation, decoction, reflux and Soxhlet extraction consume large amounts of solvents and energy. Ultrasound-assisted extraction (UAE) can utilize the mechanical, cavitation and thermal effects of ultrasonic waves to overcome the weaknesses of traditional extraction and effectively improve the extraction efficiency of flavonoids [28]. Moreover, ethanol–water system has been chosen as an extraction solvent due to its eco-friendly characteristics [29].

RSM, proposed by Box and Wilson in 1951, is an optimization method for comprehensive experimental design and mathematical modeling [30], which has been widely applied in the extraction of flavonoids from plants or herbs due to its advantages of fewer trials, higher precision and better predictive performance [31,32]. To the best of our knowledge, this study is the first attempt to simultaneously optimize UAE conditions for total flavonoid content (TFC) and their antioxidant activities (2,2-diphenyl-1-picrylhydrazyl radical scavenging capacity (DPPH-RSC) and ferric-reducing antioxidant power (FRAP)) in *A. keiskei* extracts by Box–Behnken design (BBD). Furthermore, ultrasonic temperature (X_1_), ultrasonic time (X_2_), ethanol concentration (X_3_) and liquid–solid ratio (X_4_) were selected as independent variables in order to optimize extraction process for flavonoids and antioxidant activity of *A. keiskei* employing RSM with four-factor three-level BBD.

## 2. Results and Discussion

### 2.1. Optimization of Single-Factor Experimental Extraction Conditions

#### 2.1.1. Effects of Ultrasonic Temperature on TFC

**Figure 1:**
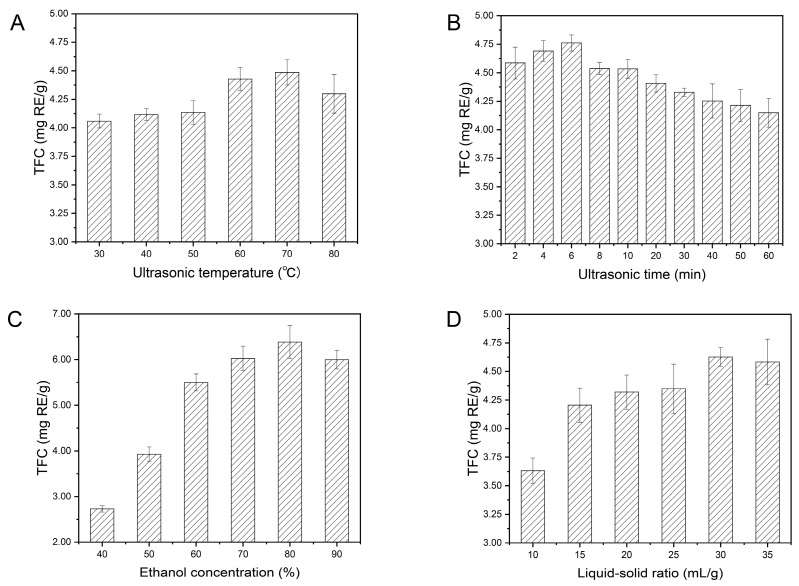
Effects of ultrasonic temperature (**A**), ultrasonic time (**B**), ethanol concentration (**C**) and liquid–solid ratio (**D**) on total flavonoid content (TFC). Results were expressed as average values ± standard deviation (*n* = 3).

It is generally accepted that the speed of molecular motion, diffusion speed, penetration and dissolution rise rapidly when ultrasonic temperature increases, which contributes to the improvement of TFC. In order to explore the effects of ultrasonic temperature on TFC, different temperatures (30 °C, 40 °C, 50 °C, 60 °C, 70 °C and 80 °C) were selected and three other factors were fixed as follows: ultrasonic time 30 min, ethanol concentration 50% (*v/v*), and liquid–solid ratio 25 mL/g. As shown in Table S1 and Figure 1A, ultrasonic temperature implied a remarkably significant effect on TFC and TFC went up slowly as the ultrasonic temperature increased continuously from 30 °C to 70 °C. However, TFC began to decrease when the temperature exceeded 70 °C. Similar phenomena were found for the extraction of flavonoid compounds from *Sophora flavescens* [33], *Fagopyrum tataricum* [34] and *Morus alba* L. Leaves [35]. Thus, it can be inferred that relative high temperatures are favorable for the denaturation of the flavonoid compounds [36,37]. Therefore, the range of ultrasonic temperature from 60 °C to 80 °C was selected for further BBD experiments.

#### 2.1.2. Effects of Ultrasonic Time on TFC

Ultrasonic time is another important element that can remarkably influence TFC. The effects of ultrasonic time (0 min to 60 min) on TFC were tested, with other conditions set as follows: ultrasonic temperature 60 °C, ethanol concentration 50% (*v/v*), and liquid–solid ratio 25 mL/g. It can be seen in Table S2 and Figure 1B that ultrasonic time showed a remarkably significant impact on TFC. When the independent variable (ultrasonic time) was changed from 2 min to 60 min, the dependent variable (TFC) reached a maximum within a short period of time (6 min), and then decreased when prolonged. The results can be mainly attributed to the following reasons: the flavonoid compounds were rapidly released in a shorter period of time from *A. keiskei* and quickly accumulated in the extraction solution. In addition, the flavonoid compounds seemed to be decomposed and emulsified; however, the flavonoids production was less than the flavonoids consumption [38,39]. Thus, it can be deductive that short-time sonication is more suitable for the extraction of flavonoids in *A. keiskei*. Therefore, an ultrasonic time within 4–8 min was selected for subsequent BBD experiments.

#### 2.1.3. Effects of Ethanol Concentration on TFC

In this study, different concentrations of ethanol solutions (40%, 50%, 60%, 70%, 80% and 90% (*v/v*)) were prepared to evaluate the effects of different ethanol concentrations on TFC, with other extraction conditions as follows: ultrasonic temperature 60 °C, ultrasonic time 30 min, and liquid–solid ratio 25 mL/g. As shown in Table S3 and Figure 1C, ethanol concentration implied a remarkably significant effect on TFC. The TFC remarkably increased with the ethanol concentration ranging from 40% to 80%, while it dropped at 90%, indicating that different ethanol–water systems possess different extraction capabilities which may be due to the variant structures of compounds and polarity of the solvent. Specifically, the maximum value of TFC was detected by extracting flavonoids with 80% ethanol, which demonstrates that the forms of flavonoids in *A. keiskei* were highly soluble in 80% ethanol solution [40,41]. Thus, the medium-high concentration of ethanol solution can be conducive to extract flavonoid compounds. Therefore, 70–90% ethanol solution was selected for further BBD experiments.

#### 2.1.4. Effects of Liquid–Solid Ratio on TFC

The liquid–solid ratio is related to the liquid–solid contact area, which can affect the extraction of flavonoids. In this study, liquid–solid ratios ranging from 10 mL/g to 35 mL/g were adopted to determine the effects of liquid–solid ratio on TFC, while other three parameters remained changeless with ultrasonic temperature at 60 °C, ultrasonic time of 30 min, ethanol concentration of 50% (*v/v*). As illustrated in Table S4 and Figure 1D, liquid–solid ratio exhibited a remarkably significant on TFC and TFC increased when the liquid–solid ratio changed from 10 mL/g to 30 mL/g. Moreover, a slowly declined trend appeared when the liquid–solid ratio exceeded 30 mL/g. These results demonstrated that freeze-drying powders of *A. keiskei* (FPAK) were fully dispersed in the ethanol solution at a high liquid–solid ratio, so that a larger contact area was obtained to enhance mass transfer. However, as the volume of ethanol increased continuously, the contact area reached saturation [42,43]. Therefore, a liquid–solid ratio range of 25–35 mL/g was chosen for further BBD experiments.

### 2.2. Model Fitting

The three-level four-factor BBD was carried out to optimize the UAE of flavonoids and antioxidant activity using RSM. Total flavonoid content (Y_TFC_), DPPH radical scavenging capacity (Y_DPPH_) and ferric-reducing antioxidant power (Y_FRAP_) acquired from 29 groups of experiments are listed in Table 1. According to previous experimental results, three second-order polynomial regression mathematical models and their mathematical expressions were automatically generated by Design-Expert software. The response variable and three fitted coding equation of the model are shown in Table 1.

The significance of the constant terms, the linear terms, the interaction terms, the square terms and the models were determined by analysis of variance (ANOVA) and the results are illustrated in Table 2. The results reveal that the three models were remarkably significant (*p* < 0.001) for TFC, DPPH-RSC and FRAP. Moreover, these two values of adjusted R² and predicted R² were very close to 1 and the value of “adjusted R²-predicted R²” was low (adjusted R²-predicted R² < 2), suggesting the regression model can fully explain the process [44,45]. In addition, “adequate precision”, as the signal-to-noise ratio, was greater than four which was desirable [46]. Furthermore, the value of the coefficient of variation for the proposed model was less than 10%, indicating the precision and reliability of the experimental run [47,48]. To conclude, it can be seen from Table 2 that the three fitted model equations conformed to the above principles and showed good adaptability.

**Figure 2:**
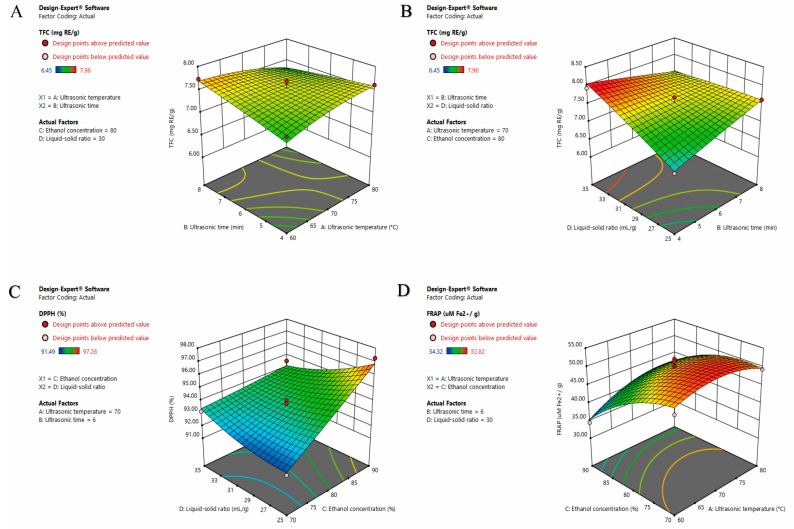
The interaction of extraction variables on TFC (**A**,**B**), DPPH-RSC (**C**) and FRAP (**D**).

In addition, a host of 3D response surface plots were generated by fixing two factors at zero level, while changing the other two factors within the scope of the exploration. These 3D graphs explored the interactive effects of the factors on TFC, DPPH-RSC and FRAP, as shown in Figure 2.

### 2.3. Effects of the Variables on TFC

The ANOVA results of TFC illustrated that the fitted Y_TFC_ model with a high *F*-value (16.16) and small *p*-value (<0.0001) was remarkably significant, as shown in Table S5 and Table 2. In addition, these results also indicated that X_3_, X_4_, X_1_X_2_, X_2_X_4_ and X_3_^2^ (*p*-value < 0.05) can significantly affect TFC while other model terms (*p*-value > 0.1) cannot. Note that the lack of fit (*F*-value = 1.18, *p*-value = 0.4747) was not remarkable compared with the pure error [49]. In other words, the non-significant lack of fit showed the Y_TFC_ model was in good agreement with the real data. In summary, this model was suitable and can be used to analyze and predict TFC in the extracts.

More specifically, the linear effect of the liquid–solid ratio (X_4_) implied a remarkably significant (*p* < 0.001) positive effect on TFC, while ethanol concentration (X_3_) and its square term (X_3_^2^) exhibited a negative one, as shown in Table S5 and Table 2. The TFC was mainly related to X_3_^2^, followed by X_2_X_4_, X_4_, X_3_ and X_1_X_2_.

It can be seen from Table S5 and Table 2 that the interaction of the ultrasonic temperature and ultrasonic time (X_1_X_2_) showed a significant (*p* < 0.05) negative effect on TFC. The TFC gradually increased at a lower ultrasonic temperature and shorter ultrasonic time. However, as displayed in Figure 2A, TFC declined with increasing ultrasonic temperature over a longer ultrasonic time. This may be caused by the acceleration of molecular movement with increasing ultrasonic temperature. Moreover, flavonoids are sensitive to temperature and decompose at a high temperature [50,51].

It can be observed from Table S5 and Table 2 that the interaction between ultrasonic time and liquid–solid ratio (X_2_X_4_) showed a remarkably significant (*p* < 0.001) negative effect on TFC. The TFC dramatically elevated with increasing liquid–solid ratio over a shorter ultrasonic time. In addition, as noted in Figure 2B, TFC showed a slowly declined trend with increasing liquid–solid ratio over a longer ultrasonic time, which can be attributed to the mechanical, cavitation and thermal effects of ultrasonic waves. Furthermore, ultrasonic waves can break the cell walls in order to accelerate the penetration of the extraction solution. However, prolonging the ultrasound time can destroy the flavonoids by the powerful energy of the ultrasonic waves [52,53].

### 2.4. Effects of the Variables on Antioxidant Activity

#### 2.4.1. Effects of the Variables on DPPH-RSC

As shown in Table S6 and Table 2, it can be clearly seen that the fitted Y_DPPH_ model showed characteristics with a high *F*-value (*F*-value = 16.16) and low *p*-value (*p*-value < 0.01), indicating that the model was remarkably significant and can be used for subsequent optimization designs. What is more, Table S6 and Table 2 suggest that X_1_, X_2_, X_3_, X_4_, X_1_X_2_, X_1_X_3_, X_1_X_4_, X_2_X_4_, X_3_X_4_ and X_4_² (*p*-value < 0.05) can significantly affect DPPH-RSC while other terms (*p*-value > 0.05) cannot. In addition, the lack of fit (*F*-value = 1.97, *p*-value = 0.2628) was non-significant, suggesting that the Y_DPPH_ model agreed well with previous experiment results. Therefore, the model is favorable to be used to analyze and predict the DPPH-RSC of extracts.

In detail, the linear effects of ultrasonic temperature (X_1_) and liquid–solid ratio (X_4_) showed significant (*p*< 0.05) negative effects on DPPH-RSC, while the linear term (ultrasonic time, X_2_) exhibited a positive one on DPPH-RSC. Moreover, the ethanol concentration (X_3_) and square term (X_4_^2^) showed remarkably significant (*p* < 0.001) and significant (*p* < 0.05) positive effects on DPPH-RSC, respectively. Furthermore, the interaction of the ultrasonic temperature and ethanol concentration (X_1_X_3_), ultrasonic temperature and liquid–solid ratio (X_1_X_4_) illustrated significant (*p* < 0.05) positive effects on DPPH-RSC, while the cross product (X_2_X_4_, ultrasonic time and liquid–solid ratio; X_1_X_2_, ultrasonic temperature and ultrasonic time) showed significant (*p* < 0.05) and remarkably significant (*p* < 0.001) negative effects on DPPH-RSC. Therefore, DPPH-RSC mainly depended on X_3_, followed by X_3_X_4_, X_1_X_2_, X_1_X_4_, X_2_X_4_, X_1_X_3_, X_4_^2^, X_2_, X_4_ and X_1_.

The interaction between the ethanol concentration and liquid–solid ratio (X_3_X_4_) showed a remarkably significant (*p* < 0.001) negative effect on DPPH-RSC (Table S6, Table 2). DPPH-RSC dramatically went up with increasing ethanol concentration. Further, at a low ethanol concentration, DPPH-RSC decreased as the liquid–solid ratio declined. However, at a high ethanol concentration, DPPH-RSC slightly increased with a decreasing liquid–solid ratio, as shown in Figure 2C.

#### 2.4.2. Effects of the Variables on FRAP

The ANOVA results of FRAP are shown in Table S7 and Table 2, which illustrated that the fitted Y_FRAP_ model with high *F*-value (16.03) and low *p*-value (*p*-value < 0.001) was remarkably significant. Furthermore, these results also implied X_1_, X_2_, X_3_, X_4_, X_1_X_3_, X_1_^2^, X_2_^2^, X_3_^2^ and X_4_² (*p*-value < 0.05) exhibited significant effects on FRAP and other model terms (*p*-value > 0.1) did not. Moreover, the lack of fit (*F*-value = 1.40, *p*-value = 0.4000) was non-significant. The results suggest that the model was suitable to be used to analyze and predict the FRAP of the extracts.

Specifically, the linear effect of X_1_, X_2_ and X_4_ illustrated highly significant (*p* < 0.01) and remarkably significant (*p* < 0.001) positive effects on FRAP, respectively, while their square terms (X_1_^2^, X_2_^2^ and X_4_^2^) exhibited highly significant (*p* < 0.01), significant (*p* < 0.05) and remarkably significant (*p* < 0.001) negative ones, respectively. In addition, the linear effect of X_3_ and its square term (X_3_^2^) exhibited a remarkably significant (*p* < 0.001) negative effect on FRAP. Furthermore, the cross product of X_1_X_3_ showed a significant (*p* < 0.05) positive effect on FRAP, as shown in Table S7 and Table 2. It can be inferred that FRAP was related to X_3_, followed by X_4_^2^, X_3_^2^, X_1_^2^, X_4_, X_1_X_3_, X_1_, X_2_^2^ and X_2_ based on these data.

The interaction between the ultrasonic temperature and ethanol concentration (X_1_X_3_) illustrated a significant (*p* < 0.05) positive effect on FRAP, as seen in Table S7 and Table 2. FRAP elevated sharply with the decrease of ethanol concentration. Further, at a low ultrasonic temperature, FRAP increased slightly as the temperature went up. However, as shown in Figure 2D, at a high ultrasonic temperature, FRAP slightly decreased with the increase of ultrasonic temperature.

### 2.5. Optimization Extraction Conditions and Verification of Predictive Model

The optimal combination of factors and levels was obtained by adopting RSM. Subsequently, the optimum extraction conditions were generated by the Design-Expert software. The extraction conditions for maximum of TFC and antioxidant activity were as follows: ultrasonic temperature 80 °C, ultrasonic time 4 min, ethanol concentration 78%, liquid–solid ratio 35 mL/g. According to the modified optimal process conditions, three repeated verification experiments were carried out. The experimental value of TFC, DPPH-RSC, FRAP and their predicted values are listed in Table 3. The experimental results were quite close to the corresponding predicted values, suggesting that the model parameters obtained by using BBD optimization were accurate and reliable.

## 3. Materials and Methods

### 3.1. Plant Materials

Freeze-drying powders of *A. keiskei* (FPAK) were supplied by Shandong Ashitaba Biotech Co., Ltd (Shandong, China), which were manufactured through a vacuum freeze-drying process (−40 °C) of fresh aerial parts of *A. keiskei* first and then was ground into powders (around 300 mesh). The powders were packed in a fresh-keeping zip lock bag and stored in a refrigerator at 4 °C before conducting the extraction experiments.

### 3.2. Chemicals and Reagents

Sodium hydroxide was purchased from Guangfu Technology Development Co., Ltd. (Tianjin, China). Hydrochloric acid was obtained from Sanhe Chemical Reagent Co., Ltd. (Yantai, China). Sodium acetate anhydrous was acquired from Guangcheng Chemical Reagent Co., Ltd. (Tianjin, China). 2,2-Diphenyl-1-picrylhydrazyl (DPPH, 96% purity) and 2,4,6-tris(2-pyridyl)-s-triazine (TPTZ, 99% purity) were supplied by RHAWN Chemical Reagent Co., Ltd. (Shanghai, China). Ethanol and acetic acid were bought from Fuyu Fine Chemical Co., Ltd. (Tianjin, China). Aluminum nitrate nonahydrate and rutin hydrate (analytical reference) were purchased from Macklin Biochemical Co., Ltd. (Shanghai, China). Sodium nitrite, iron (Ⅲ) chloride hexahydrate and iron (Ⅱ) sulfate heptahydrate were supplied by Sinopharm Chemical Reagent Co., Ltd. (Shanghai, China). Distilled water was provided by Chengda Distilled Water Co., Ltd. (Qingdao, China). All other chemicals and reagents used in this study were of analytical reagent grade.

### 3.3. Equipment for UAE

**Figure 3:**
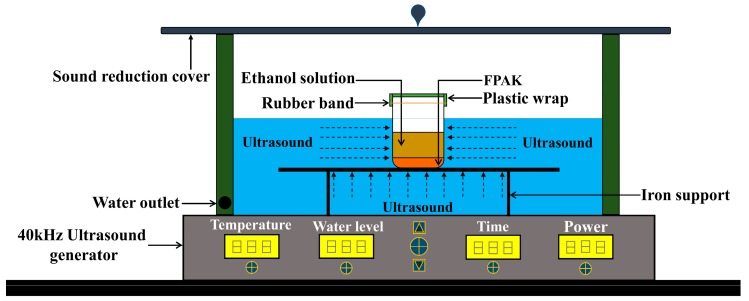
Diagram of experimental ultrasonic-assisted extraction device.

The scheme of experimental setup for the extraction process is depicted in Figure 3. For the UAE experiments, a digitally-controlled ultrasonic cleaning machine (KQ-300DE, Kunshan Ultrasonic Instrument Co., Ltd., Jiangsu, China) with fixed frequency at 40 kHz was adopted as the ultrasound generator. FPAK were placed in a 100 mL beaker containing ethanol solution, and then the beaker mouth was sealed with polyethylene plastic wrap and a rubber band. Finally, a tiny hole in the plastic wrap was pierced by the disposable medical injection needle to prevent the plastic wrap from exploding due to excessive pressure inside the beaker.

### 3.4. UAE of Flavonoids from A. keiskei

Firstly, FPAK were placed into a 100 mL beaker, and steeped in ethanol solution at a certain concentration in a given ratio of liquid to solid, then the beaker was placed into the digitally-controlled ultrasonic cleaning machine with a fixed frequency of 40 kHz at a given time and temperature. After the extraction, the flask was removed from the bath. Subsequently, the extracts were finely filtered with filter paper and transferred to a volumetric flask. Then s specific concentration of ethanol solution was cautiously added until a precisely calibrated final volume was reached. The solution was shaken till evenly mixed. Finally, the filtrates were collected in a 100 mL hermetically sealed plastic bottle and kept in a refrigerator at 4 °C until further experimental analysis. 

### 3.5. Single-Factor Experiments

In this part, single-factor experiments were performed to explore the effects of the following parameters on TFC: ultrasonic temperature (30–80 °C), ultrasonic time (0–60 min), ethanol concentration (40–90%) and liquid–solid ratio (10–35 mL/g). According to the single-factor experimental data, the maximum values in these four sets of experiments were the optimal central values for TFC. A series of studies were carried out afterwards to determine the best extraction process of *A. keiskei* by using the RSM.

### 3.6. BBD for Extraction Optimization

According to single-factor experiments, a three-level four-factor BBD with RSM was conducted to optimize the UAE of flavonoids and antioxidant activity in FPAK. Ultrasonic temperature (°C, X_1_), ultrasonic time (min, X_2_), ethanol concentration (%, X_3_) and liquid–solid ratio (mL/g, X_4_) were selected as the main influencing factors. Based on the results of previous single-factor experiments, TFC was regarded as an important indicator for determining the range of each independent variable. Independent variables and their codes and levels used for RSM are shown in Table 4. The BBD contained 29 groups of experiments which are listed in Table 5.

A second-order polynomial regression mathematical model was used to express the value of Y_TFC_, Y_DPPH_ and Y_FRAP_. The mathematical model was shown in Equation (1).

(1) $$Y=\beta_{0}+\overset{4}{\underset{j=1}{\sum }}\beta_{j}X_{j}+\overset{4}{\underset{j=1}{\sum }}\beta_{\mathrm{jj}}X_{j}^{2}+\overset{3}{\underset{i=1}{\sum }}\overset{4}{\underset{j=i+1}{\sum }}\beta_{\mathrm{ij}}X_{i}X_{j}$$

where Y is the response variable; β_0_ is a fixed value that means the intercept of the model; β_j_, β_jj_ and βij are the linear, quadratic and interactive coefficients, respectively; X_i_ and X_j_ represent the coded level of independent variables.

### 3.7. Determination of Total Flavonoid Content (TFC)

The TFC from *A. keiskei* in extracts was determined by a NaNO_2_-Al(NO_3_)_3_-NaOH method described in two reports with a few adjustments [54,55]. Briefly, the extracts were firstly centrifuged at 8000 rpm for 10 min, and the upper-layer solution was collected for further detection of TFC. One mL of the solution was transferred into a 10 mL volumetric flask with the addition of 0.4 mL 5% (*w/v*) NaNO_2_ solution afterwards. Six minutes later, 0.4 mL of 10% (*w/v*) Al(NO_3_)_3_ was added and the mixture stood for 6 min. Subsequently, 4.0 mL of 5% (*w/v*) NaOH solution was added into the volumetric flask to form a 10 mL solution with distilled water. Then the solution was mixed thoroughly and incubated for 30 min at room temperature. Finally, the absorbance of the solution was measured immediately against a blank at 510 nm using a UV–Vis spectrophotometer (TU-1901, Persee General Instrument Co., Ltd., Beijing, China). Rutin was used as the standard for a calibration curve and TFC was expressed as rutin equivalent (RE) per gram of FPAK (mg RE/g) and calculated by Equation (2).

(2) $$\mathrm{TFC}=\frac{\mathrm{The}\;\mathrm{flavonoids}\;\mathrm{content}\;\mathrm{of}\;\mathrm{extracts}\;\left(\mathrm{mg}\;\mathrm{RE}\right)}{\mathrm{weight}\;\mathrm{of}\;\mathrm{FPAK}\;\left(g\right)}$$

### 3.8. Determination of Antioxidant Activities

#### 3.8.1. DPPH Radical Scavenging Capacity (DPPH-RSC) Measurement

DPPH-RSC was measured using a colorimetric method described by Wang et al. with certain modifications [56]. Firstly, the extracts were centrifuged at 8000 rpm for 10 min, and the upper-layer solution was collected for determination of DPPH-RSC. Briefly, 2.5 mL of 0.1 mM DPPH ethanol solution and 2.5 mL of the upper-layer solution were thoroughly mixed. Then the mixture was incubated for 30 min at room temperature in the dark. At last, the absorbance A_1_ was measured immediately against a blank (absolute ethanol) at 517 nm. For the blank control, 2.5 mL of absolute ethanol and 2.5 mL of extract was mixed. This mixture was kept still in the dark for 30 min, and the absorbance A_2_ was measured at 517 nm with absolute ethanol as the blank. For negative control, 2.5 mL of 0.1 mM DPPH and 2.5 mL of absolute ethanol was mixed. Then the mixture was placed in the dark for 30 min, and the absorbance A_0_ was measured at 517 nm with absolute ethanol as a blank. DPPH-RSC was calculated by Equation (3):

(3) $$\mathrm{DPPH}-\mathrm{RSC}\;\left(\%\right)=\left[\frac{A_{0}-\left(A_{1}-A_{2}\right)}{A_{0}}\right]\times 100$$

#### 3.8.2. Ferric-Reducing Antioxidant Power (FRAP) Assay

FRAP was determined by a colorimetric method described by Impei et al. with some modifications [57]. The extracts were centrifuged at 8000 rpm for 10 min, and the upper-layer solution was collected. A fresh FRAP reagent was prepared by mixing 25 mL of 20 mM FeCl_3_ꞏ6H_2_O solution with 25 mL of 10 mM TPTZ solution (40 mM HCl solution as the solvent), and 250 mL of 300 mM acetate buffer containing 5.1 g CH_3_COONa and 20 mL CH_3_COOH (pH = 3.6). Briefly, 3.9 mL freshly prepared FRAP reagent and 130 μL upper-layer solution was mixed. Then the reaction mixture was incubated at 37 °C for 15 min in the dark. Lastly, the absorbance of the solution was measured immediately at 593 nm against a reagent blank (3.9 mL of FRAP solution and 130 μL of distilled water). The FRAP was expressed as FeSO_4_ equivalents per gram of FPAK (μmol Fe^2+^/g) and calculated as Equation (4) through the calibration curve of FeSO_4_. In Equation (4), C was the Fe^2+^ concentration (μmol Fe^2+^/L) corresponding to the absorbance of the sample solution, V was the total volume of the sample solution (L), and W was the mass of FPAK (g).

(4) $$\mathrm{FRAP}=\frac{C\;\times \;V}{W}$$

### 3.9. Statistical Analysis

All extraction experiments, TFC determination experiments and antioxidant activity assays were carried out in triplicates. All results were expressed as average values ± standard deviation (*n* = 3) and analyzed by OriginPro 2018 (OriginLab Corporation, Northampton, MA, USA), SPSS statistics v25.0 (IBM Corporation, New York, NY, USA) and Design-Expert v11.0 (Stat-Ease Inc., Minneapolis, MN, USA). To determine the individual linear, quadratic and interaction regression coefficients (β), ANOVA was performed. The fitness of the polynomial was estimated by employing the coefficient of determination (R^2^), and the significance of each coefficient was determined by *p*-values. Specifically, *p*-value ≤ 0.001, 0.001 < *p*-value ≤ 0.01, 0.01< *p*-value ≤ 0.05 and *p*-value > 0.05 indicate that the model terms are remarkably significant, highly significant, significant and not significant, respectively [58].

## 4. Conclusions

In this study, single-factor experiments were carried out to determine the optimal extraction parameters under different conditions. A three-level four-factor BBD was performed to explore the linear, cross and quadratic effects by using RSM of the following parameters on flavonoids and antioxidant activity: ultrasonic temperature (X_1_), ultrasonic time (X_2_), ethanol concentration (X_3_) and liquid–solid ratio (X_4_). Experimental results indicated that X_3_ and X_4_ affected TFC, DPPH-RSC and FRAP. Furthermore, X_1_ and X_2_ also had an influence on the DPPH-RSC and FRAP, while X_1_ and X_2_ had little correlation with TFC. In addition, X_2_X_4_, X_3_^2^; X_1_X_2_, X_3_X_4_ and X_3_^2^, X_4_^2^ showed a remarkably significant effect on TFC, DPPH-RSC and FRAP, respectively. The optimum conditions of the combination of TFC, DPPH-RSC and FRAP were as follows: X_1_ = 80 °C, X_2_ = 4 min, X_3_ = 78% and X_4_ = 35 mL/g. Based on three repeated verification experiments, it was found that the experimental results were quite close to the corresponding predicted values. These results can provide a theoretical basis for the comprehensive utilization of *A. keiskei* and the extraction of its flavonoids as a potential source of antioxidants. In addition, further analyses and comprehensive researches should be warranted for clarifying the main bioactive components and their contents in the extracts of *A. keiskei*, and the mechanisms of their bioactivities still need to be explored.

## Figures and Tables

**Table 1:** Response variables and their fitted model equations.

Symbol	Response Variable	Fitting the Coding Equation of the Model
Y_TFC_	Total flavonoid content (mg RE/g)	Y_TFC_ = 7.5459 + 0.0014X_1_ + 0.0529X_2_−0.2517X_3_ + 0.2763X_4_ − 0.1827X_1_X_2_ − 0.0065X_1_X_3_ + 0.0837X_1_X_4_ + 0.0992X_2_X_3_ − 0.3285X_2_X_4_ + 0.0278X_3_X_4_ − 0.0796X_1_² − 0.0099X_2_² − 0.5671X_3_² − 0.0266X_4_²
Y_DPPH_	DPPH radical scavenging capacity (%)	Y_DPPH_ = 93.6219 − 0.2886X_1_ + 0.3209X_2_ + 1.4200X_3_ − 0.3100X_4_ − 0.8693X_1_X_2_ + 0.4834X_1_X_3_ + 0.6182X_1_X_4_ − 0.0165X_2_X_3_ − 0.5440X_2_X_4_ − 1.0414X_3_X_4_ + 0.0552X_1_² + 0.2049X_2_² + 0.0356X_3_² + 0.4082X_4_²
Y_FRAP_	Ferric-reducing antioxidant power (μM Fe^2+^/g)	Y_FRAP_ = 49.6304 + 1.7734X_1_ + 1.6391X_2_ − 5.3144X_3_ + 2.2654X_4_ − 1.4726X_1_X_2_ + 1.8035X_1_X_3_ + 0.0422X_1_X_4_ + 0.5681X_2_X_3_ + 0.2908X_2_X_4_ + 1.3159X_3_X_4_ − 2.6215X_1_² − 1.6820X_2_² − 2.7125X_3_² − 3.1732X_4_²

X_1_: ultrasonic temperature (°C); X_2_: ultrasonic time (min); X_3_: ethanol concentration (%); X_4_: liquid–solid ratio (mL/g). DPPH: diphenyl-1-picrylhydrazyl.

**Table 2:** Regression coefficient (β) and fit statistics of the predicted second-order polynomial models for flavonoids and antioxidant activity.

Factor	Coefficient (β)		
	TFC	DPPH-RSC	FRAP
Intercept	7.5459	93.6219	49.6304
Linear			
X_1_	0.0014	−0.2886 *	1.7734 **
X_2_	0.0529	0.3209 *	1.6391 **
X_3_	−0.2517 ***	1.4200 ***	−5.3144 ***
X_4_	0.2763 ***	−0.3100 *	2.2654 ***
Cross product			
X_1_X_2_	−0.1827 *	−0.8693 ***	−1.4726
X_1_X_3_	−0.0065	0.4834 *	1.8035 *
X_1_X_4_	0.0837	0.6182 *	0.0422
X_2_X_3_	0.0992	−0.0165	0.5681
X_2_X_4_	−0.3285 ***	−0.5440 *	0.2908
X_3_X_4_	0.0278	−1.0414 ***	1.3159
Quadratic			
X_1_²	−0.0796	0.0552	−2.6215 **
X_2_²	−0.0099	0.2049	−1.6820 *
X_3_²	−0.5671 ***	0.0356	−2.7125 ***
X_4_²	−0.0266	0.4082 *	−3.1732 ***
R²	0.9417	0.9427	0.9413
Adjusted R²	0.8835	0.8854	0.8825
Predicted R²	0.7264	0.7106	0.7166
Adequate precision	16.3886	17.7803	14.0570
Coefficient of variation	1.95%	0.4427%	3.65%
*p*-Value (Model)	<0.0001 ***	<0.0001 ***	<0.0001 ***
*p*-Value (Lack of fit)	0.4747	0.2682	0.4000

X_1_: ultrasonic temperature (°C); X_2_: ultrasonic time (min); X_3_: ethanol concentration (%); X_4_: liquid–solid ratio (mL/g). *: indicates significance level (0.01 < *p*-value ≤ 0.05); **: indicates highly significant level (0.001 < *p*-value ≤ 0.01); ***: indicates remarkably significant level (*p*-value ≤ 0.001). TFC: total flavonoid content; FRAP: ferric-reducing antioxidant power; DPHH-RSC: 2,2-diphenyl-1-picrylhydrazyl radical scavenging capacity.

**Table 3:** Experimental values and predicted values of response variables at optimum extraction conditions.

Response Variables	Optimum Extraction Conditions ^1^				Maximum Value	
	X_1_	X_2_	X_3_	X_4_	Experimental Value ^2^	Predicted Value
Y_TFC_ (mg RE/g)	80 °C	4 min	78%	35 mL/g	7.96 ± 0.18	8.29
Y_DPPH_ (%)					94.68 ± 0.57	95.25
Y_FRAP_ (μM Fe^2+^/g)					45.35 ± 0.23	46.17

^1^ X_1_: ultrasonic temperature (°C); X_2_: ultrasonic time (min); X_3_: ethanol concentration (%); X_4_: liquid–solid ratio (mL/g). ^2^ Experimental results were expressed as average values ± standard deviation (*n* = 3).

**Table 4:** Independent variables and their levels in Box–Behnken design (BBD).

Independent Variable	Symbol	Level		
		−1	0	1
Ultrasonic temperature (°C)	X_1_	60	70	80
Ultrasonic time (min)	X_2_	4	6	8
Ethanol concentration (%)	X_3_	70	80	90
Liquid-solid ratio (mL/g)	X_4_	25	30	35

**Table 5:** Designed experiments and measured responses of response surface analysis.

Run	Ultrasonic Temperature	Ultrasonic Time	Ethanol Concentration	Liquid-Solid Ratio	Response 1-Y_TFC_	Response 2-Y_DPPH_	Response 3-Y_FRAP_
	(℃)	(min)	(%)	(mL/g)	(mg RE/g)	(%)	(μM Fe^2+^/g)
1	60	6	70	30	6.95	93.09	47.97
2	80	8	80	30	7.27	92.88	45.92
3	70	6	80	30	7.52	93.29	48.20
4	60	6	80	35	7.73	93.55	45.93
5	80	6	70	30	7.00	91.49	49.38
6	60	8	80	30	7.74	94.92	45.66
7	80	6	80	35	7.96	94.00	48.43
8	60	6	80	25	7.14	95.25	41.82
9	70	4	70	30	7.36	92.15	50.65
10	80	6	80	25	7.04	93.22	44.15
11	70	6	80	30	7.43	93.36	48.06
12	60	4	80	30	7.34	93.36	40.36
13	70	4	80	25	6.79	93.47	39.37
14	80	4	80	30	7.61	94.79	46.51
15	70	8	80	25	7.62	95.70	42.96
16	60	6	90	30	6.68	94.92	34.54
17	70	6	70	35	7.47	93.16	49.11
18	70	6	80	30	7.68	93.80	51.47
19	70	6	90	35	6.93	94.53	40.19
20	70	4	80	35	7.93	93.81	44.95
21	70	6	80	30	7.69	94.06	50.24
22	70	6	70	25	7.10	91.73	48.50
23	70	8	70	30	7.28	93.14	52.82
24	70	8	90	30	6.84	95.38	43.45
25	70	6	80	30	7.41	93.60	50.17
26	80	6	90	30	6.71	95.25	43.16
27	70	6	90	25	6.45	97.26	34.32
28	70	4	90	30	6.52	94.46	39.01
29	70	8	80	35	7.44	93.87	49.70

## Supplementary Materials

The following are available online; Tables S1–S7.

## References

[1] Zhang C., Liu D., Gao H. (2018). Kinetics, physicochemical properties, and antioxidant activities of *Angelica keiskei* processed under four drying conditions. LWT Food Sci. Technol..

[2] Carmona-Gutierrez D., Zimmermann A., Kainz K., Pietrocola F., Chen G., Maglioni S., Schiavi A., Nah J., Mertel S., Beuschel C.B. (2019). The flavonoid 4,4’-dimethoxychalcone promotes autophagy-dependent longevity across species. Nat. Commun..

[3] Kil Y.S., Choi S.K., Lee Y.S., Jafari M., Seo E.K. (2015). Chalcones from *Angelica keiskei*: Evaluation of Their Heat Shock Protein Inducing Activities. J. Nat. Prod..

[4] Zhang L., Li Z., Wang L., Gao X., Li Q. (2018). Research Progress of Anti-tumor Effects of Chalcones from *Angelica keiskei*. Sci. Technol. Food Ind..

[5] Nakamura T., Tokushima T., Kawabata K., Yamamoto N., Miyamoto M., Ashida H. (2012). Absorption and metabolism of 4-hydroxyderricin and xanthoangelol after oral administration of *Angelica keiskei* (Ashitaba) extract in mice. Arch. Biochem. Biophys..

[6] Li J.L., Gao L.X., Meng F.W., Tang C.L., Zhang R.J., Li J.Y., Luo C., Li J., Zhao W.M. (2015). PTP1B inhibitors from stems of *Angelica keiskei* (Ashitaba). Bioorg. Med. Chem. Lett..

[7] Zhang W., Jin Q., Luo J., Wu J., Wang Z. (2018). Phytonutrient and anti-diabetic functional properties of flavonoid-rich ethanol extract from *Angelica keiskei* leaves. J. Food Sci. Technol..

[8] Xie F., Wang Y., Zhou Y., Wu J., Wang Z. (2017). Effect of lactic acid bacteria on microbial safety of *angelica keiskei* juice. J. Food Saf..

[9] Sarker S.D., Nahar L. (2004). Natural medicine: The genus Angelica. Curr. Med. Chem..

[10] Zhang T., Yamashita Y., Yasuda M., Yamamoto N., Ashida H. (2015). Ashitaba (*Angelica keiskei*) extract prevents adiposity in high-fat diet-fed C57BL/6 mice. Food Funct..

[11] Nikitakis J., Lange B. (2016). International Cosmetic Ingredient Dictionary and Handbook.

[12] Chen C.Y. (2004). Trace elements in Taiwanese health food, *Angelica keiskei*, and other products. Food Chem..

[13] Cao X., Zhang M., Mujumdar A.S., Wang Z. (2019). Effect of microwave freeze-drying on microbial inactivation, antioxidant substance and flavor quality of Ashitaba leaves (*Angelica keiskei* Koidzumi). Dry. Technol..

[14] Sugamoto K., Matsusita Y.I., Matsui K., Kurogi C., Matsui T. (2011). Synthesis and antibacterial activity of chalcones bearing prenyl or geranyl groups from *Angelica keiskei*. Tetrahedron.

[15] Park J.Y., Jeong H.J., Kim Y.M., Park S.J., Rho M.C., Park K.H., Ryu Y.B., Lee W.S. (2011). Characteristic of alkylated chalcones from *Angelica keiskei* on influenza virus neuraminidase inhibition. Bioorg. Med. Chem. Lett..

[16] Hagiwara H., Nakata K., Miyazaki H., Maehashi S., Komiyama Y., Aida R., Yoshida S., Kokubu D., Hagiwara K., Yoshida K. (2019). 4-Hydroxyderricin inhibits osteoclast formation and accelerates osteoblast differentiation. Cytotechnology.

[17] Li Z., Zhang L., Gao M., Han M., Liu K., Zhang Z., Gong Z., Xing L., Shi X., Lu K. (2019). Endoplasmic reticulum stress triggers Xanthoangelol-induced protective autophagy via activation of JNK/c-Jun Axis in hepatocellular carcinoma. J. Exp. Clin. Cancer Res..

[18] Ogawa H., Ohno M., Baba K. (2005). Hypotensive and lipid regulatory actions of 4-hydroxyderricin, a chalcone from *Angelica keiskei*, in stroke-prone spontaneously hypertensive rats. Clin. Exp. Pharmacol. Physiol..

[19] Yasuda M., Kawabata K., Miyashita M., Okumura M., Yamamoto N., Takahashi M., Ashida H., Ohigashi H. (2014). Inhibitory effects of 4-hydroxyderricin and xanthoangelol on lipopolysaccharide-induced inflammatory responses in RAW264 macrophages. J. Agric. Food Chem..

[20] Ohnogi H., Kudo Y., Tahara K., Sugiyama K., Enoki T., Hayami S., Sagawa H., Tanimura Y., Aoi W., Naito Y. (2012). Six new chalcones from *Angelica keiskei* inducing adiponectin production in 3T3-L1 adipocytes. Biosci. Biotechnol. Biochem..

[21] Kil Y.S., Pham S.T., Seo E.K., Jafari M. (2017). *Angelica keiskei*, an emerging medicinal herb with various bioactive constituents and biological activities. Arch. Pharmacal Res..

[22] Borges Bubols G., da Rocha Vianna D., Medina-Remon A., von Poser G., Maria Lamuela-Raventos R., Lucia Eifler-Lima V., Cristina Garcia S. (2013). The Antioxidant Activity of Coumarins and Flavonoids. Mini Rev. Med. Chem..

[23] Agati G., Azzarello E., Pollastri S., Tattini M. (2012). Flavonoids as antioxidants in plants: Location and functional significance. Plant Sci..

[24] Yao L.H., Jiang Y., Shi J., Tomas-Barberan F., Datta N., Singanusong R., Chen S. (2004). Flavonoids in food and their health benefits. Plant Foods Hum. Nutr..

[25] Halliwell B. (1994). Free radicals, antioxidants, and human disease: Curiosity, cause, or consequence?. Lancet.

[26] Pham-Huy L.A., He H., Pham-Huy C. (2008). Free radicals, antioxidants in disease and health. Int. J. Biomed. Sci..

[27] Oludemi T., Barros L., Prieto M.A., Heleno S.A., Barreiro M.F., Ferreira I. (2018). Extraction of triterpenoids and phenolic compounds from *Ganoderma lucidum*: Optimization study using the response surface methodology. Food Funct..

[28] Cai X., Zhang R., Guo Y., He J., Li S., Zhu Z., Liu G., Liu Z., Yang J. (2015). Optimization of ultrasound-assisted extraction of gardenia fruit oil with bioactive components and their identification and quantification by HPLC-DAD/ESI-MS^2^. Food Funct..

[29] Farrell A.E., Plevin R.J., Turner B.T., Jones A.D., Michael O.H., Kammen D.M. (2006). Ethanol can contribute to energy and environmental goals. Science.

[30] Box G.E.P., Wilson K.B. (1951). On the Experimental Attainment of Optimum Conditions. J. R. Stat. Soc. Ser. B (Methodol.).

[31] Briones-Labarca V., Giovagnoli-Vicuna C., Canas-Sarazua R. (2019). Optimization of extraction yield, flavonoids and lycopene from tomato pulp by high hydrostatic pressure-assisted extraction. Food Chem..

[32] Agarwal C., Mathe K., Hofmann T., Csoka L. (2018). Ultrasound-Assisted Extraction of Cannabinoids from *Cannabis Sativa* L. Optimized by Response Surface Methodology. J. Food Sci. Technol..

[33] Zhou J., Zhang L., Li Q., Jin W., Chen W., Han J., Zhang Y. (2018). Simultaneous Optimization for Ultrasound-Assisted Extraction and Antioxidant Activity of Flavonoids from *Sophora flavescens* Using Response Surface Methodology. Molecules.

[34] Peng L.X., Zou L., Zhao J.L., Xiang D.B., Zhu P., Zhao G. (2013). Response surface modeling and optimization of ultrasound-assisted extraction of three flavonoids from tartary buckwheat (*Fagopyrum tataricum*). Pharmacogn. Mag..

[35] Cui H., Lu T., Wang M., Zou X., Zhang Y., Yang X., Dong Y., Zhou H. (2019). Flavonoids from *Morus alba* L. Leaves: Optimization of Extraction by Response Surface Methodology and Comprehensive Evaluation of Their Antioxidant, Antimicrobial, and Inhibition of alpha-Amylase Activities through Analytical Hierarchy Process. Molecules.

[36] Sheng Z.L., Wan P.F., Dong C.L., Li Y.H. (2013). Optimization of total flavonoids content extracted from *Flos Populi* using response surface methodology. Ind. Crop. Prod..

[37] Wang Y., Gao Y., Ding H., Liu S., Han X., Gui J., Liu D. (2017). Subcritical ethanol extraction of flavonoids from *Moringa oleifera* leaf and evaluation of antioxidant activity. Food Chem..

[38] Lai J., Wang H., Wang D., Fang F., Wang F., Wu T. (2014). Ultrasonic extraction of antioxidants from Chinese sumac (*Rhus typhina* L.) fruit using response surface methodology and their characterization. Molecules.

[39] Tomaz I., Maslov L., Stupić D., Preiner D., Ašperger D., Karoglan Kontić J. (2016). Multi-response optimisation of ultrasound-assisted extraction for recovery of flavonoids from red grape skins using response surface methodology. Phytochem. Anal..

[40] Xu D.P., Zheng J., Zhou Y., Li Y., Li S., Li H.B. (2017). Ultrasound-assisted extraction of natural antioxidants from the flower of *Limonium sinuatum*: Optimization and comparison with conventional methods. Food Chem..

[41] Tian J., Muhammad S., Chen A., Chen P., Wang J., Yang C., Yuan H., Wang Z. (2019). An experimental study exploring the influencing factors for ultrasonic-assisted extraction of flavonoid compounds from leaves of *Amorpha fruticosa* L.. J. For. Res..

[42] Zhang Y., Prawang P., Li C., Meng X., Zhao Y., Wang H., Zhang S. (2018). Ultrasonic assisted extraction of artemisinin from *Artemisia Annua* L. using monoether-based solvents. Green Chem..

[43] Tabaraki R., Nateghi A. (2011). Optimization of ultrasonic-assisted extraction of natural antioxidants from rice bran using response surface methodology. Ultrason. Sonochem..

[44] Li F., Mao Y.D., Wang Y.F., Raza A., Qiu L.P., Xu X.Q. (2017). Optimization of Ultrasonic-Assisted Enzymatic Extraction Conditions for Improving Total Phenolic Content, Antioxidant and Antitumor Activities in Vitro from *Trapa quadrispinosa* Roxb. Residues. Molecules.

[45] Chen W., Wang W.P., Zhang H.S., Huang Q. (2012). Optimization of ultrasonic-assisted extraction of water-soluble polysaccharides from *Boletus edulis* mycelia using response surface methodology. Carbohydr. Polym..

[46] Erbay Z., Icier F. (2009). Optimization of Drying of Olive Leaves in a Pilot-Scale Heat Pump Dryer. Dry. Technol..

[47] Chen G., Chen J., Srinivasakannan C., Peng J. (2012). Application of response surface methodology for optimization of the synthesis of synthetic rutile from titania slag. Appl. Surf. Sci..

[48] Eslami A., Asadi A., Meserghani M., Bahrami H. (2016). Optimization of sonochemical degradation of amoxicillin by sulfate radicals in aqueous solution using response surface methodology (RSM). J. Mol. Liq..

[49] Kostić M.D., Bazargan A., Stamenković O.S., Veljković V.B., McKay G. (2016). Optimization and kinetics of sunflower oil methanolysis catalyzed by calcium oxide-based catalyst derived from palm kernel shell biochar. Fuel.

[50] Wang B., Qu J., Luo S., Feng S., Li T., Yuan M., Huang Y., Liao J., Yang R., Ding C. (2018). Optimization of Ultrasound-Assisted Extraction of Flavonoids from Olive (*Olea europaea*) Leaves, and Evaluation of Their Antioxidant and Anticancer Activities. Molecules.

[51] Zhu C.P., Zhai X.C., Li L.Q., Wu X.X., Li B. (2015). Response surface optimization of ultrasound-assisted polysaccharides extraction from pomegranate peel. Food Chem..

[52] Lin T., Liu Y., Lai C., Yang T., Xie J., Zhang Y. (2018). The effect of ultrasound assisted extraction on structural composition, antioxidant activity and immunoregulation of polysaccharides from *Ziziphus jujuba* Mill var. spinosa seeds. Ind. Crop. Prod..

[53] Shirzad H., Niknam V., Taheri M., Ebrahimzadeh H. (2017). Ultrasound-assisted extraction process of phenolic antioxidants from Olive leaves: A nutraceutical study using RSM and LC-ESI-DAD-MS. J. Food Sci. Technol..

[54] Wang X., Wu Q., Wu Y., Chen G., Yue W., Liang Q. (2012). Response surface optimized ultrasonic-assisted extraction of flavonoids from Sparganii rhizoma and evaluation of their *in vitro* antioxidant activities. Molecules.

[55] Zhang G., He L., Hu M. (2011). Optimized ultrasonic-assisted extraction of flavonoids from *Prunella vulgaris* L. and evaluation of antioxidant activities in vitro. Innov. Food Sci. Emerg. Technol..

[56] Wang S., Dong X., Tong J. (2013). Optimization of enzyme-assisted extraction of polysaccharides from alfalfa and its antioxidant activity. Int. J. Biol. Macromol..

[57] Impei S., Gismondi A., Canuti L., Canini A. (2015). Metabolic and biological profile of autochthonous *Vitis vinifera* L. ecotypes. Food Funct..

[58] Chen S., Zeng Z., Hu N., Bai B., Wang H., Suo Y. (2018). Simultaneous optimization of the ultrasound-assisted extraction for phenolic compounds content and antioxidant activity of *Lycium ruthenicum* Murr. fruit using response surface methodology. Food Chem..

