# Supplementary material for: Simultaneous Optimization of Ultrasound-Assisted Extraction for Flavonoids and Antioxidant Activity of Angelica keiskei Using Response Surface Methodology (RSM)
Source: Molecules. 2019 Sep 24;24(19):3461. doi: 10.3390/molecules24193461 (PMC6804174; doi:10.3390/molecules24193461)
Supplement: Supplementary file 1 [file molecules-24-03461-s001.pdf]

molecules

MDPI

Supplementary Information

Simultaneous Optimization of Ultrasound-Assisted Extraction for Flavonoids and Antioxidant Activity of *Angelica keiskei* Using Response Surface Methodology (RSM)

Lei Zhang [1,2], Yuhuan Jiang [1], Xuening Pang [1], Puyue Hua [1], Xiang Gao [1], Qun Li [2,*] and Zichao Li [1,2,3,*]

[1] College of Life Sciences, Institute of Advanced Cross-Field Science, Institute of *Angelica keiskei* Health Industry Technology, Qingdao University, Qingdao 266071, China; 2017020832@qdu.edu.cn (L.Z.); 2018025210@qdu.edu.cn (Y.J.); 2018025216@qdu.edu.cn (X.P.); h13399545149@163.com (P.H.); gaoxiang@qdu.edu.cn (X.G.)

[2] College of Chemistry and Chemical Engineering, Qingdao University, Qingdao 266071, China;

[3] Qingdao Balanson Biotech Co., Ltd., Qingdao 266071, China

* Correspondence:zichaoli@qdu.edu.cn (Z.L.); qunli@qdu.edu.cn (Q.L.); Tel.: +86-532-8595-0705 (Q.L.)

Table S1 Analysis of variance (ANOVA) for ultrasonic temperature on the TFC.

Source	Sum of squares	Degree of freedom	Mean square	*F*-value	*p*-value	Significant [1]
Ultrasonic temperature	0.472	5	0.094	8.280	0.001	***
Error	0.137	12	0.011			
Total	0.609	17				

[1] *: indicate significant level (0.01 < *p*-value ≤ 0.05); **: indicate highly significant level (0.001 < *p*-value ≤ 0.01); ***: indicate remarkably significant level (*p*-value ≤ 0.001).

Table S2 ANOVA for ultrasonic time on the TFC.

Source	Sum of squares	Degree of freedom	Mean square	*F*-value	*p*-value	Significant [1]
Ultrasonic time	1.169	9	0.130	12.171	<0.0001	***
Error	0.213	20	0.011			
Total	1.382	29				

[1] *: indicate significant level (0.01 < *p*-value ≤ 0.05); **: indicate highly significant level (0.001 < *p*-value ≤ 0.01); ***: indicate remarkably significant level (*p*-value ≤ 0.001).

Table S3 ANOVA for ethanol concentration on the TFC.

Source	Sum of squares	Degree of freedom	Mean square	*F*-value	*p*-value	Significant [1]
Ethanol concentration	31.368	5	6.274	123.324	<0.0001	***
Error	0.610	12	0.051			
Total	31.978	17				

[1] *: indicate significant level (0.01 < *p*-value ≤ 0.05); **: indicate highly significant level (0.001 < *p*-value ≤ 0.01); ***: indicate remarkably significant level (*p*-value ≤ 0.001).

Table S4 ANOVA for liquid-solid ratio on the TFC.

Source	Sum of squares	Degree of freedom	Mean square	*F*-value	*p*-value	Significant [1]
Liquid-solid ratio	1.932	5	0.386	15.403	<0.0001	***
Error	0.301	12	0.025			
Total	2.234	17				

[1] *: indicate significant level (0.01 < *p*-value ≤ 0.05); **: indicate highly significant level (0.001 < *p*-value ≤ 0.01); ***: indicate remarkably significant level (*p*-value ≤ 0.001).

Table S5 ANOVA for the fitted quadratic polynomial model of TFC.

Source [1]	Sum of squares	Degree of freedom	Mean square	*F*-value	*p*-value	Significant [2]
Y_{TFC}-Model	4.53	14	0.3232	16.16	< 0.0001	***
X_1	2.3593×10^{-5}	1	2.3593×10^{-5}	0.0012	0.9731	
X_2	0.0336	1	0.0336	1.68	0.2157	
X_3	0.7601	1	0.7601	38.00	< 0.0001	***
X_4	0.9162	1	0.9162	45.81	< 0.0001	***
X_1X_2	0.1335	1	0.1335	6.68	0.0216	*
X_1X_3	0.0002	1	0.0002	0.0084	0.9285	
X_1X_4	0.0280	1	0.0280	1.40	0.2561	
X_2X_3	0.0394	1	0.0394	1.97	0.1825	
X_2X_4	0.4316	1	0.4316	21.58	0.0004	***
X_3X_4	0.0031	1	0.0031	0.1551	0.6997	
X_1^2	0.0411	1	0.0411	2.06	0.1735	
X_2^2	0.0006	1	0.0006	0.0319	0.8609	
X_3^2	2.09	1	2.09	104.30	< 0.0001	***
X_4^2	0.0046	1	0.0046	0.2288	0.6398	
Residual	0.2800	14	0.0200			
Lack of fit	0.2090	10	0.0209	1.18	0.4747	
Pure error	0.0710	4	0.0178			
Cor total	4.81	28				

[1] X_1: ultrasonic temperature (℃); X_2: ultrasonic time (min); X_3: ethanol concentration (%); X_4: liquid-solid ratio (mL/g).
[2] *: indicate significant level (0.01 < *p*-value ≤ 0.05); **: indicate highly significant level (0.001 < *p*-value ≤ 0.01); ***: indicate remarkably significant level (*p*-value ≤ 0.001).

Table S6 ANOVA for the fitted quadratic polynomial model of DPPH-RSC.

Source [1]	Sum of squares	Degree of freedom	Mean square	*F*-value	*p*-value	Significant [2]
Y_{DPPH}-Model	39.82	14	2.84	16.46	< 0.0001	***
X_1	0.9998	1	0.9998	5.78	0.0306	*
X_2	1.24	1	1.24	7.15	0.0182	*
X_3	24.20	1	24.20	139.99	< 0.0001	***
X_4	1.15	1	1.15	6.67	0.0217	*
X_1X_2	3.02	1	3.02	17.49	0.0009	***
X_1X_3	0.9346	1	0.9346	5.41	0.0356	*

X_1X_4	1.53	1	1.53	8.84	0.0101	*
X_2X_3	0.0011	1	0.0011	0.0063	0.9379	
X_2X_4	1.18	1	1.18	6.85	0.0203	*
X_3X_4	4.34	1	4.34	25.10	0.0002	***
X_1^2	0.0198	1	0.0198	0.1143	0.7403	
X_2^2	0.2725	1	0.2725	1.58	0.2298	
X_3^2	0.0082	1	0.0082	0.0475	0.8307	
X_4^2	1.08	1	1.08	6.25	0.0254	*
Residual	2.42	14	0.1728			
Lack of fit	2.01	10	0.2012	1.97	0.2682	
Pure error	0.4082	4	0.1021			
Cor total	42.24	28				

[1] X_1: ultrasonic temperature (℃); X_2: ultrasonic time (min); X_3: ethanol concentration (%); X_4: liquid-solid ratio (mL/g).
[2] *: indicate significant level (0.01 < *p*-value ≤ 0.05); **: indicate highly significant level (0.001 < *p*-value ≤ 0.01); ***: indicate remarkably significant level (*p*-value ≤ 0.001).

Table S7 ANOVA for the fitted quadratic polynomial model of FRAP.

**Source** [1]	**Sum of squares**	**Degree of freedom**	**Mean square**	***F*-value**	***p*-value**	**Significant** [2]
Y_{FRAP}-Model	617.51	14	44.11	16.03	< 0.0001	***
X_1	37.74	1	37.74	13.71	0.0024	**
X_2	32.24	1	32.24	11.71	0.0041	**
X_3	338.92	1	338.92	123.14	< 0.0001	***
X_4	61.58	1	61.58	22.37	0.0003	***
X_1X_2	8.67	1	8.67	3.15	0.0976	
X_1X_3	13.01	1	13.01	4.73	0.0473	*
X_1X_4	0.0071	1	0.0071	0.0026	0.9602	
X_2X_3	1.29	1	1.29	0.4690	0.5046	
X_2X_4	0.3382	1	0.3382	0.1229	0.7311	
X_3X_4	6.93	1	6.93	2.52	0.1350	
X_1^2	44.58	1	44.58	16.20	0.0013	**
X_2^2	18.35	1	18.35	6.67	0.0217	*
X_3^2	47.73	1	47.73	17.34	0.0010	***
X_4^2	65.32	1	65.32	23.73	0.0002	***
Residual	38.53	14	2.75			
Lack of fit	29.96	10	3.00	1.40	0.4000	
Pure error	8.58	4	2.14			
Cor total	656.05	28				

[1] X_1: ultrasonic temperature (℃); X_2: ultrasonic time (min); X_3: ethanol concentration (%); X_4: liquid-solid ratio (mL/g).
[2] *: indicate significant level (0.01 < *p*-value ≤ 0.05); **: indicate highly significant level (0.001 < *p*-value ≤ 0.01); ***: indicate remarkably significant level (*p*-value ≤ 0.001).
